# Perceived self-efficacy and empowerment in patients at increased risk of sudden cardiac arrest

**DOI:** 10.3389/fcvm.2023.955060

**Published:** 2023-05-15

**Authors:** Brianna Davies, Katherine S. Allan, Sandra L. Carroll, Karen Gibbs, Jason D. Roberts, Ciorsti MacIntyre, Christian Steinberg, Rafik Tadros, Paul Dorian, Jeff S. Healey, Martin Gardner, Zachary W. M. Laksman, Andrew D. Krahn, Anne Fournier, Colette Seifer, Sandra B. Lauck

**Affiliations:** ^1^ Centre for Cardiovascular Innovation, St. Paul’s and Vancouver General Hospitals, University of British Columbia, Vancouver, BC, Canada; ^2^ Division of Cardiology, St. Michael’s Hospital, University of Toronto, Toronto, ON, Canada; ^3^ School of Nursing, Faculty of Health Science, Population Health Research Institute, McMaster University, Hamilton, ON, Canada; ^4^ Section of Cardiac Electrophysiology, Division of Cardiology, Department ofMedicine, Western University, London, ON, Canada; ^5^ QEII Health Sciences Center, Halifax, NS, Canada; ^6^ Institut Universitaire de Cardiologie et Pneumologie de Québec, Laval University, Quebec City, QC, Canada; ^7^ Department of Medicine, Cardiovascular Genetics Center, Montreal Heart Institute, Université de Montréal, Montreal, QC, Canada; ^8^ Division of Pediatric Cardiology, CHU Sainte-Justine, Université de Montréal, Montreal,QC, Canada; ^9^ Department of Internal Medicine, St Boniface Hospital, University of Manitoba, Winnipeg, MB, Canada

**Keywords:** cardiogenetics, genetic counselling, self efficacy, empowerment, patient engagement

## Abstract

**Background:**

The role of multidisciplinary clinics for psychosocial care is increasingly recognized for those living with inherited cardiac conditions (ICC). In Canada, access to healthcare providers differ between clinics. Little is known about the relationship between access to specialty care and a patient's ability to cope with, and manage their condition.

**Methods:**

We leveraged the Hearts in Rhythm Organization (HiRO) to conduct a cross-sectional, community-based survey of individuals with ICC and their family members. We aimed to describe access to services, and explore the relationships between participants’ characteristics, cardiac history and self-reported health status and self-efficacy (GSE: General Self-Efficacy Scale) and empowerment (GCOS-24: Genetic Counseling Outcome Scale).

**Results:**

We collected 235 responses from Canadian participants in 10 provinces and territories. Overall, 63% of participants reported involvement of a genetic counsellor in their care. Access to genetic testing was associated with greater empowerment [mean GCOS-24: 121.14 (SD = 20.53) vs. 105.68 (SD = 21.69); *p* = 0.004]. Uncertain genetic test results were associated with lower perceived self-efficacy (mean GSE: uncertain = 28.85 vs. positive = 33.16, negative = 34.13; *p *= 0.01). Low global mental health scores correlated with both lower perceived self-efficacy and empowerment scores, with only 11% of affected participants reporting involvement of psychology services in their care.

**Conclusion:**

Differences in resource accessibility, clinical history and self-reported health status impact the perceived self-efficacy and empowerment of patients with ICC. Future research evaluating interventions to improve patient outcomes is recommended.

## Introduction

Inherited cardiac conditions (ICC) include, long QT syndrome (LQTS), short QT syndrome (SQTS) arrhythmogenic cardiomyopathies (ARVC/ACM), catecholaminergic polymorphic ventricular tachycardia (CPVT), early repolarization syndromes (ERS) and Brugada syndrome (BrS). These conditions can result in sudden unexpected death, typically in a seemingly healthy child or young adult before the condition can be recognized and treated (1–3). In recent years, advances in genomic technologies have markedly improved the ability to identify those at risk for premature sudden cardiac death due to an arrhythmia, and facilitated the implementation of preventive strategies.

The psychological impact of undergoing screening and/or living with an ICC is also increasingly recognized, with past research demonstrating high levels of patient anxiety, depression and poor adjustment to their diagnosis (4–8). Identifying those struggling to adapt and cope with their ICC is important, particularly given that low self-efficacy and low empowerment are known barriers to engage in risk prevention strategies, such as medication adherence or screening recommendations, critical for reducing risk of sudden cardiac death (9, 10). Further, a positive correlation between empowerment and uptake of cardiac screening in first-degree relatives has been reported (11). Together, this growing evidence underlines the importance of implementing strategies to improve patient self-efficacy and empowerment in cardiogenetic care delivery.

Expert consensus guidelines for the management of families with ICC have highlighted the importance of multidisciplinary clinics, including access to genetic counsellors and psychology resources, to support families cope with and manage the psychological impacts of ICCs (12–15).

In contrast to these recent recommendations, families report differences in both delivery of cardiogenetics care and access to providers across Canada. While access to specialty ICC clinics is covered under the public health system, these clinics are typically located within major urban centres, and the logistics and/or cost of travel for families in rural areas can be a significant barrier to accessing care providers. Further, not all specialty ICC clinics in Canada have a genetic counsellor embedded within the cardiology department, with some requiring patients to be referred to different clinics for a separate genetic counselling appointment, which can often have long waitlists.

Little is known regarding the relationship between access to care services and a patient's ability to cope and manage their ICC. We conducted a cross-sectional survey to explore relationships between patient characteristics, health status and access to care services with perceived self-efficacy and empowerment of ICC patients and their family members in Canada. The main objectives of this study were to (1) understand the current state of care provider access for patients with ICC in Canada and (2) explore relationships between access to certain care providers and perceived self-efficacy and empowerment. Secondary objectives included establishing baseline measures of self-efficacy and empowerment in the ICC population and exploring sub-populations with lower self-efficacy and empowerment to aid in the design of future interventions to improve patient-reported outcomes for ICC patients and their relatives.

## Methods

This study was approved by the UBC Providence Health Care research ethics board (H17-01894).

### Recruitment and community engagement

The National Hearts in Rhythm Organization (HiRO) is a group of clinicians, researchers, patients and families working together to improve detection and treatment of inherited arrhythmia and cardiomyopathy disorders in Canada (16). In addition to leading a national research registry, HiRO has numerous working groups dedicated to improving clinical care and supporting patient advocacy efforts. We leveraged this national network to form the HiRO Patient-Oriented Research Working Group, bringing together members interested in working alongside patient partners to conduct research to contribute novel evidence and improve psychosocial outcomes for patients living with ICC in Canada. Patient partners co-led framework development, study design and funding applications of this first working group project.

Patients with an ICC or unexplained cardiac arrest, their first-degree family members and their caregivers over the age of 18, were invited to complete an anonymous survey administered electronically through a university-affiliated online survey tool. Participants were recruited from families followed at nine HiRO clinics in five provinces across Canada. We provided clinical teams with business cards to distribute to families containing the online survey link at the time of their clinic visit. Additionally, families who were already participating in the National HiRO Registry [including Cardiac Arrest Survivors with Preserved Ejection Fraction Registry (CASPER), National ARVC registry and National Long QT Syndrome registry] at these centers who had previously consented to be re-contacted for research purposes received a letter of invitation. The online survey link was also available from the HiRO website (www.heartsinrhythm.org) and social media accounts (@heartsinrhythm). Additionally, the Canadian Sudden Arrhythmogenic Death Syndrome (SADS) Foundation partnered with the HiRO research team to promote survey recruitment on their online platforms, and increase the awareness of the survey outside of major care centers.

#### Study framework and survey design

**Figure 1:**
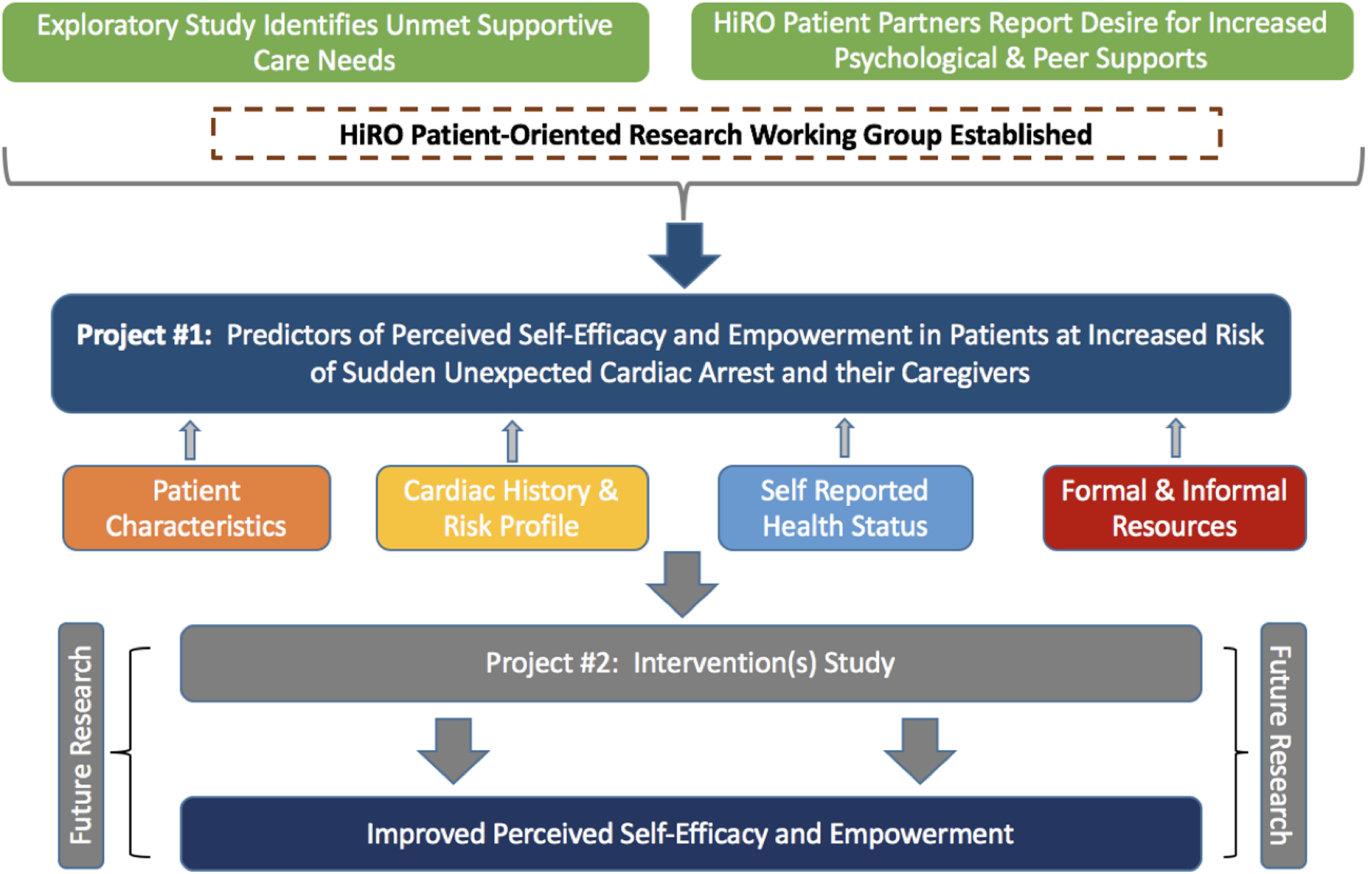
Framework of the HiRO patient-oriented research working group.

This study was grounded in a framework developed by the HiRO Patient-Oriented Research Group based on current evidence, clinician expertise and the input of people with lived experience. We hypothesized four domains (personal demographics, cardiac history and risk profile, self-reported health status, and resource accessibility) that drive patients’ capacity for self-efficacy and empowerment (Figure 1).

The constructs of general self-efficacy and empowerment were selected as key patient-reported outcome measures for the psychological well-being of ICC patients and their relatives. Both have previously been associated with health-related quality of life and adherence to risk-prevention measures, making these outcomes well-suited to measure impact of future interventions (9–11, 17–21). Self-efficacy is defined as the belief that one can perform a novel or difficult task, or cope with adversity (22). Self-efficacy is considered a key component of patient self-management of their disease, and has been found to positively correlate with self-esteem and optimism and negatively correlate with anxiety (22). While there have been differing definitions of patient empowerment in the literature, most agree that it relates to a patient's ability to take control of their wellbeing, play an active role in their healthcare and integrate their diagnosis within their self-identity (23, 24). Measures of patient empowerment have been found to correlate positively with constructs of perceived personal control, and decisional conflict and negatively with depression (25). While some have previously described an empowered patient as one who has “mastered” self-efficacy, there is general consensus that measures of self-efficacy fail to capture the broader psychosocial components of patient empowerment, particularly the emotional and cognitive domains (23, 24, 26). In 2018, a study by Kohler at el evaluated both patient empowerment and general self-efficacy in patients with coronary heart disease, and found the relationship between the two variables to be weak (*r* = 0.38) (27). The study concluded that patient empowerment and general self-efficacy are not interchangeable and should both be taken into consideration with designing healthcare to maximize health-related quality of life (27).

##### Survey design

An electronic survey (Supplementary Appendix S1) was designed to collect candidate variables in each of these four predicted domains, and was available in both English and French. Patient demographics, cardiac history and risk profile (including history of cardiac arrest & ICD shocks), and access to resources were captured by a survey designed by the research team. Self-reported health status was collected from three validated measures: 10-item Global Health PROMIS scale, 4-item Anxiety PROMIS 4a Scale, and 12-item Multidimensional Scale of Perceived Social Support (MSPSS). The Global Health PROMIS Scale is a measure of overall health-related quality of life which generates a physical and emotional health score with high internal reliability scores (*r* = 0.81 and 0.86) (28–31). Participants’ emotional distress was captured with the Anxiety PROMIS 4a Scale, the short form version that is well correlated (*r* = 0.90) to the extensively validated PROMIS Anxiety 8a (*α* = 0.93; *r* = 0.79) (32). Lastly, the domain of social health status was measured with the Multidimensional Scale of Perceived Social Support (MSPSS), designed to assess global perceived support from family, friends and significant others. This validated measure has good internal reliability (*α* = 0.85–0.91) and factor analysis between each sub-group has been shown to correlate with different sources of support (33–35). Each of the PROMIS and MSPSS scales have been validated in both English and French.

Participants’ level of confidence in managing their condition was measured using the General Self-Efficacy Scale (GSE), which has strong internal reliability and concurrent validity with numerous positive emotions and optimism and has been validated in both English and French (17, 36). Lastly, the Genetic Counselling Outcome Scale (GCOS-24) was used to measure patient empowerment (25). In this study, empowerment was defined based on the contributions of McAllister et al. as the belief that one can make important life decisions (decisional control), has sufficient information about their family's condition (cognitive control), can manage one's feelings (behavioural control), can make effective use of the healthcare system (emotional regulation), and has hope for the future (hope) (25). The scale has been shown to have high internal consistency, test-retest reliability and construct validity with the measurement of health locus of control, satisfaction with life and depression. While this scale has yet to be validated in French, the feasibility of translation and cultural consistency has been demonstrated by recent validation of the scale in both Danish and Spanish populations (37, 38). The English version of this survey was professionally translated to French and reviewed by a bilingual study team member for use in this study.

### Statistical analysis

Categorical variables are reported as total responses and percentages. Continuous variables are listed as means and standard deviations. Each of the validated instruments were scored according to the reference scoring guides to provide an overall score (15–18).

Part 1: Demographic variables are reported as total response and percentages (Table 1). Single variable analysis was performed to identify the relationship between each predictor variable and general self-efficacy and empowerment. Unpaired t-tests were used to compare mean outcome scores between those who reported access to each care provider versus those who did not (Table 2). Analysis of variance (ANOVA) tests were performed to test the interaction between cardiac history variables with more than two groups with mean outcome scores reported. Tukey HSD tests were performed if ANOVA was significant (Supplementary Tables S1–S6). Bivariable linear regression was performed to identify the relationship between self-reported health status scores and outcome variables (Table 3 and Supplementary Table S7). For all statistical tests, level of significance was considered 0.05 and *t*-tests were one-tailed.

**Table 1:** Demographics of HiRO survey participants.

Demographics	*n* (%)
**Responses**	235
**Survey language**	
English	226 (96%)
French	9 (4%)
**Sex**	
Female	156 (66%)
Male	75 (32%)
Other	1 (0%)
No answer	3 (1%)
**Age**	
18–34	37 (16%)
35–54	96 (41%)
55 and up	72 (31%)
No Answer	30 (12%)
**Province/Territory**	
Western Canada	115 (49%)
Prairies	29 (12%)
Ontario	56 (24%)
Quebec	19 (8%)
Eastern Canada	14 (6%)
No answer	1 (0%)
**Primary language**	
English	202 (86%)
Another language	25 (11%)
No answer	8 (3%)
**Highest education**	
High school	25 (11%)
Some post-secondary	83 (35%)
Undergraduate degree or more	122 (52%)
Other	1 (0%)
No answer	4 (2%)
**Main activity**	
Employed	158 (67%)
Not employed	68 (29%)
Other	7 (3%)
No answer	2 (1%)
**Annual household income**	
Less than $69,999	65 (28%)
More than $70,000	143 (61%)
No answer	27 (11%)
**Relationship status**	
In a relationship	178 (76%)
Not in a relationship	48 (20%)
No answer	9 (4%)

**Table 2:** Self-efficacy and empowerment scores by health care provider (HCP) accessed for ICC care.

HCP Accessed for ICC care:	Affected (*n* = 160)							Unaffected relative/partner (*n* = 51)						
	*n* (%)	Mean GSE score (SD)			Mean GCOS score (SD)			*n* (%)	Mean GSE score (SD)			Mean GCOS score (SD)		
		YES	NO	*p*	YES	NO	*p*		YES	NO	*p*	YES	NO	*p*
Heart rhythm specialist	152 (95%)	32.87 (5.36)	29.25 (5.70)	0.12	118.71 (20.52)	111.89 (30.68)	0.53	30 (59%)	30.89 (6.05)	34.41 (3.61)	0.02*	114.33 (9.81)	120.00 (24.22)	0.46
Genetic counsellor	100 (63%)	32.96 (5.17)	32.18 (5.85)	0.42	120.46 (20.47)	114.35 (22.29)	0.12	19 (37%)	30.22 (6.63)	33.56 (4.21)	0.07	114.76 (19.87)	117.33 (22.59)	0.70
Psychologist	17 (11%)	30.92 (7.55)	32.86 (5.15)	0.37	114.92 (18.86)	118.61 (21.56)	0.52	4 (8%)	22.67 (13.01)	32.90 (4.12)	0.31	94.67 (18.61)	117.97 (20.74)	0.15
Family doctor	116 (73%)	32.41 (5.71)	33.39 (4.51)	0.29	117.82 (22.16)	119.36 (18.96)	0.69	22 (43%)	30.45 (6.59)	33.64 (4.03)	0.07	116.25 (18.44)	116.29 (24.15)	0.99
Pharmacist	45 (28%)	32.52 (5.38)	32.73 (5.47)	0.83	119.26 (17.87)	117.82 (22.62)	0.70	3 (6%)	23.50 (19.09)	32.63 (4.38)	0.62	123.00 (36.77)	115.92 (20.95)	0.83
Social worker	11 (7%)	32.40 (7.21)	32.69 (5.30)	0.90	111.80 (14.39)	118.77 (21.70)	0.18	5 (10%)	26.75 (3.60)	32.76 (5.38)	0.03*	100.25 (17.84)	118.00 (21.10)	0.14
Physical therapist	10 (6%)	29.00 (6.24)	32.94 (5.29)	0.08	110.50 (19.50)	118.88 (21.36)	0.22	0 (0%)	NA	32.22 (5.49)	NA	NA	116.27 (21.29)	NA
Pediatrician	3 (2%)	29.33 (5.13)	32.74 (5.43)	0.37	103.67 (12.22)	118.58 (21.35)	0.16	5 (10%)	33.00 (4.08)	32.15 (5.65)	0.72	120.75 (19.47)	115.78 (21.67)	0.66
Trauma counsellor	6 (4%)	35.33 (4.13)	32.56 (5.46)	0.17	113.17 (9.97)	118.48 (21.66)	0.27	1 (2%)	31.00 (NA)	32.25 (5.55)	NA	144.00 (NA)	115.58 (21.08)	NA
Research coordinator	41 (26%)	32.23 (5.50)	32.84 (5.41)	0.55	120.53 (21.16)	117.32 (21.36)	0.43	10 (20%)	31.00 (3.81)	32.53 (5.84)	0.35	114.22 (11.38)	116.84 (3.45)	0.64

HCP, healthcare provider; ICC, inherited cardiogenetic condition; GSE, general self-efficacy score; GCOS, genetic counselling outcome score. Six affected participants did not answer which HCPs they accessed for ICC care and were excluded from this analysis.

* Denotes statistical significance (*p* < 0.05).

Part 2: Predictor variables identified to have a *p*-value less than or equal 0.2 on single variable analysis for each outcome variable were then entered into a multiple linear regression model for each general self-efficacy and patient empowerment. Separate models were calculated for (1) affected patients (Tables 4, 5) and (2) unaffected relatives and/or caregivers (Supplementary Tables S8, S9). For each model, coefficients and 95% confidence intervals were reported.

**Table 3:** Self-efficacy and empowerment scores by self-reported health status in affected patients.

Self-reported health status:	General self-efficacy (GSE)					Patient empowerment (GCOS-24)				
	*B*	95% CI		*F*-Statistic	*p*	*B*	95% CI		*F*-Statistic	*p*
Global health—physical	−0.78	−2.50	0.95	0.788	0.38	−4.51	−11.59	2.57	1.59	0.21
Global health—mental	4.69	3.32	6.07	45.45	<0.001*	14.57	8.38	20.75	21.73	<0.001*
Perceived social support	1.20	0.63	1.76	17.36	<0.001*	4.69	2.16	7.22	13.43	<0.001*

* Denotes statistical significance (*p* < 0.05).

**Table 4:** Multiple linear regression model for general self-efficacy (gse): affected patients.

Affected patients (*n* = 125):		*B*	95% CI	
Highest education: (*High School*)	Some post-secondary education	1.68	−1.17	4.52
	Bachelor's degree or higher	2.26	−0.57	5.09
*Main activity* (*Employed or Student*)	Not currently employed	−0.78	−2.53	0.98
Relationship (*Not in a relationship*)	In a relationship	1.39	−1.07	3.84
Income (*No Answer*)	Under $69,000 per year	2.26	−1.19	5.72
	More than $70,000 per year	2.83	−0.59	6.25
Healthcare providers	Heart rhythm speciality	3.16	−0.19	6.51
	Physical therapist	−3.24	−6.34	−0.13
	Trauma counsellor	2.66	−1.24	6.56
GT result: (*Not applicable*)	Positive	−0.58	−2.56	1.41
	Negative	−0.13	−2.63	2.38
	The results were unclear	−3.52	−6.60	−0.44
Exercise restrictions: (*No restrictions*)	Yes—Worried about cardiac risk	−3.95	−6.32	−1.58
	Yes—Physical limitations	1.31	−2.66	5.27
	Yes—Healthcare provider recommendations	−1.33	−5.30	2.64
	Yes—Other	1.20	−0.90	3.30
ICD Shock	Yes (No)	−1.32	−3.88	1.23
Hx anxiety or depression	Yes (No)	−0.22	−2.04	1.60
Global health—mental		2.44	0.74	4.15
MSPSS		−0.01	−0.66	0.65

Variables in brackets denotes the variable used as reference. GT, genetic testing; MSPSS, multidimensional scale of perceived social support. 41 of 166 affected participants did not provide an answer for at least one of the predictor variables are were excluded from this analysis (*n* = 125).

All statistical tests were performed in R-statistic (Version 1.2.1335). Survey participants who selected “no answer” or did not provide a response were excluded from analysis utilizing those variables. Only participants who provided answers to each of the applicable predictor variables were included in the multivariable analyses.

## Results

In total, 235 survey responses were completed between January 2018 and March 2021 (Table 1). Incomplete data for one of GSE or GCOS scores was reported in 59 cases (25.1%). Overall, 66% of respondents identified as female, 41% were between the ages of 34–54 years, and 86% of participants reported English as their primary language.

**Table 5:** Multiple linear regression model for patient empowerment (GCOS-24): affected patients.

Affected patients (*n* = 115)		*B*	95% CI	
Highest education: *(High School)*	Some post-secondary education	5.66	−6.78	18.10
	Bachelor's degree or higher	10.37	−2.07	22.81
*Main activity (Employed or Student)*	Not currently employed	−4.32	−12.73	4.09
Relationship *(Not in a relationship)*	In a relationship	5.82	−4.18	15.83
Income *(No answer)*	Under $69,000 per year	−0.49	−23.27	22.30
	More than $70,000 per year	−0.13	−22.76	22.50
Healthcare providers	Genetic counsellor	−2.88	−12.67	6.92
	Social worker	6.92	−7.60	21.43
	Pediatrician	−7.74	−34.66	19.18
Genetic testing	Yes (No)	16.41	5.99	26.82
Exercise restrictions: *(No restrictions)*	Yes—Worried about cardiac risk	−25.25	−34.85	−15.64
	Yes—Physical limitations	−1.47	−19.77	16.83
	Yes—Healthcare provider recommendations	−3.58	−18.24	11.08
	Yes—Other	1.32	−8.70	11.33
Sudden cardiac arrest	Yes (No)	−4.94	−15.13	5.26
ICD	Yes (No)	4.67	−4.61	13.94
Shocks	Yes (No)	−8.51	−20.65	3.64
Hx anxiety or depression	Yes (No)	−1.46	−9.58	6.66
Global health—mental		11.37	3.90	18.85
MSPSS		0.53	−2.45	3.52

Variables in brackets denotes the variable used as reference. MSPSS, multidimensional scale of perceived social support. 51 of 166 affected participants did not provide an answer for at least one of the predictor variables are were excluded from this analysis (*n* = 115).

166 (71%) respondents had a personal diagnosis of an ICC or experienced an unexplained cardiac arrest, 44 (19%) were unaffected first-degree relatives, 7 (3%) identified as a spouse/partner or close friend, and 18 (7%) declined to answer this question (Supplementary Table S1).

### Part 1

Participant’s education, main activity, relationship status and income were associated with statistically significant differences for both general self-efficacy (GSE) and patient empowerment (GCOS-24).

Overall, unaffected first-degree relatives and partner/spouses of someone with an ICC had similar mean perceived self-efficacy (GSE) and empowerment (GCOS-24) compared to those living with a diagnosis [GSE: 28.51 (SD = 3.58) (unaffected relative) vs. 29.17 (SD = 1.17) (partner/spouse) vs. 28.52 (SD = 2.62) (affected proband) vs. 28.93 (SD = 2.46) (affected relatives); *p* = 0.80]; [GCOS: 115.53 (SD = 20.98) (unaffected relative) vs. 121.60 (SD = 25.32) (partner/spouse) vs. 117.91 (SD = 21.09) (affected proband) vs. 118.71 (SD = 21.71) (affected relative); *p* = 0.81] (Supplementary Table S1). There were no statistically significant differences in general self-efficacy or patient empowerment in those with a different ICC diagnoses (Supplementary Table S2).

#### Access to healthcare providers

Among participants with a personal diagnosis of an ICC, 95% (*n* = 152) reported access to a heart rhythm specialist and 63% (*n* = 100) had access to a genetic counsellor (Table 2). The majority of participants had access to both these care providers (59%; *n* = 98), whereas 36% (*n* = 59) had access to a heart rhythm specialist only; 2 participants (1%) had access to a genetic counsellor only and 4% (*n* = 7) reported access to neither. A lower proportion of unaffected first-degree relatives or partners reported access to a heart rhythm specialist (59%; *n* = 30) or genetic counsellor (37%; *n* = 19). Access to psychologists was low amongst both affected patients and their relatives/partners [affected:11% (*n* = 17); relatives: 8% (*n* = 4)].

Overall, affected participants whose ICC team included a heart rhythm specialist reported greater self-efficacy scores (GSE) [32.87 (SD = 5.36) vs. 29.25 (SD = 5.70); *p *= 0.12] whereas those who reported access to a genetic counsellor had greater empowerment scores [120.46 (SD = 20.47) vs. 114.35 (SD = 22.29); *p* = 0.12], however neither of these findings were statistically significant (Table 2). In unaffected relatives, those who reported access to a heart rhythm specialist had significantly lower self-efficacy scores [heart rhythm specialist: mean GSE: 30.89 (SD = 6.05) vs. 34.41 (SD = 3.61); *p *= 0.02].

#### Clinical history

Affected participants who had genetic testing performed reported significantly higher empowerment (GCOS-24), regardless of the findings, compared to those who were not tested [121.14 (SD = 20.53) vs. 105.68 (SD = 21.69); *p *= 0.004] (Supplementary Table S3). However, participants with an unclear genetic test result (i.e., variant of uncertain significance) had lower self-efficacy scores compared to those with either a positive [28.85 (SD = 5.32) (VUS) vs. 33.16 (SD = 5.31) (positive); *p* = 0.04] or negative genetic test result [28.85 (SD = 5.32) (VUS) vs. 34.13 (SD = 4.40) (negative); *p* = 0.02] (Supplementary Table S4). In unaffected relatives/spouses, those whose family members had genetic testing performed also had higher empowerment scores, however this was not statistically significant [118.22 (SD = 22.08) vs. 103.71 (SD = 18.27); *p* = 0.22] (Supplementary Table S6).

Affected participants who restricted exercise based on their ICC diagnosis had significantly lower empowerment (GCOS-24) scores compared to those whose exercise habits were not changed [112.03 (SD = 21.22) vs. 123.76 (SD = 19.90) *p* = 0.001] (Supplementary Table S3). Participants who reported self-restricting exercise due to worry it may increase risk of cardiac arrest or ICD shock had significantly lower empowerment (GCOS-24) scores compared to those who reduce exercise due to physical limitations [95.71 (SD = 15.07) vs. 117.86 (SD = 17.86); *p* = 0.03], healthcare provided advice [95.71 (SD = 15.07) vs. 124.75 (SD = 14.11); *p* = <0.001] or other reasons [95.71 (SD = 15.07) vs. 121.32 (SD = 20.95); *p* = <0.001] (Supplementary Table S5). Affected participants with a prior history of anxiety or depression prior to receiving a diagnosis of an ICC had both lower perceived self-efficacy [31.20 (SD = 6.06) vs. 33.58 (SD = 4.92); *p* = 0.01] and empowerment scores [114.30 (SD = 22.59) vs. 120.54 (SD = 20.39); *p* = 0.05] (Supplementary Table S3). Additionally, unaffected relatives/partners whose family member experienced a sudden cardiac death reported lower empowerment scores [109.21 (SD = 20.33) vs. 123.65 (SD = 21.00); *p* = 0.04] (Supplementary Table S6).

#### Self-reported health status

Lower scores on both the mental health component of the PROMIS Scale v1.2—Global Health survey and the Multidimensional Scale of Perceived Social Support (MSPSS) were associated with lower perceived self-efficacy (GSE) (Global Health—Mental: *B* = 4.69, *p *= <0.001; MSPSS: *B* = 1.20, *p *= <0.001) and empowerment (GCOS-24) (Global Health—Mental: *B* = 14.57, *p* = <0.001; MSPSS: *B* = 4.69; *p *= <0.001) in affected participants (Table 3). In unaffected relatives/partners, higher scores on the mental health component of the PROMIS Scale v1.2—Global Health survey was associated with higher self-efficacy (*B* = 5.32; *p* = <0.001), whereas higher scores on the Multidimensional Scale of Perceived Social Support (MSPSS) were associated with higher empowerment (*B* = 5.64; *p* = 0.01) (Supplementary Table S7).

### Part 2

All variables identified in Part 1 to correlate with general self-efficacy (GSE) or patient empowerment (GCOS-24) at a significance level of 0.2 or less were then entered to multivariable linear regression models for both affected patients (Tables 4, 5) and unaffected relatives/partners (Supplementary Tables S8, S9).

#### Affected patients

In the multiple linear regression model, uncertain genetic test results (VUS) (*B* = −3.52), exercise restrictions due to worry about cardiac risk (*B* = −3.95) and PROMIS Global Health—Mental Health scores (*B* = 2.44) were correlated with general self-efficacy (Table 4). Access to genetic testing (*B* = 16.41), exercise restrictions due to worry about cardiac risk (*B* = −25.25) and PROMIS Global Health—Mental Health scores (*B* = 11.37) were correlated with patient empowerment (Table 5).

#### Unaffected relatives and partners

PROMIS Global Health—Mental Health scores (*B* = 5.69) correlated with general self-efficacy in unaffected relatives or partners (Supplementary Table S8).

## Discussion

This study makes novel contributions to better understand the needs of individuals and families affected by ICC. Our unique focus on exploring the complex drivers of living well with ICC provide innovative insights to strengthen patient-oriented research. We identified clinical history and self-reported health status variables associated with general self-efficacy and empowerment in a cohort of patients at increased risk of sudden death. In partnership with people with lived experience and community partners, we sought to capture diverse perspectives. Access to specialized care, including availability of genetic testing was associated with higher patient empowerment, whereas receiving uncertain genetic test results was associated with lower general self-efficacy. Lower self-reported mental health scores were associated with lower perceived self-efficacy and empowerment. Despite lower cardiac risk profiles, no significant differences in self-efficacy and empowerment scores were found between affected patients and their unaffected relatives.

### Access to speciality care

Overall, 95% of ICC patients reported access to a heart rhythm specialist and 63% reported access to a genetic counsellor, with 59% reporting access to both healthcare professionals. Genetic counselling for inherited cardiac disorders has previously been associated with greater patient empowerment (39). Our study identified a similar trend, with mean patient empowerment scores being greater in affected patients who reported access to a genetic counsellor, although this was not statistically significant (*p *= 0.12). The benefits of receiving multidisciplinary care in specialized cardiogenetics clinics is also becoming increasingly recognized, including improved access to genetic testing, identified to be a predictor of greater patient empowerment in this study (13, 39–41). In this model of health service delivery, genetic counsellors may also be involved in the care of patients over time, allowing for the development of a therapeutic relationship and greater involvement in psychosocial care after initial diagnosis. Recently, a study by Murray et al. reported an association between strength of genetic counsellor-patient relationship and patient empowerment, further supporting the added value of this model of care (42). Continued efforts should be made to improve access to both genetic counselling and genetic testing for ICC patients in Canada *via* the establishment of multidisciplinary clinics. Barriers to creating multidisciplinary clinics in Canada include identifying funding to support genetic counsellor salary, in addition to recruitment challenges given the small genetic counsellor workforce.

### Clinical history and risk profiles

Multiple studies have previously explored patients’ motivations to pursue genetic testing in the cardiac context, with a common theme being the desire to reduce uncertainty surrounding their diagnosis (43, 44). However, for those whose result includes a variant of uncertain significance (VUS), the finding may instead add to the burden of uncertainty. A study by Predham et al., evaluated patients’ perspective of receiving inconclusive genetic test results in LQTS, and highlighted that some patients were disappointed by the lack of conclusive findings. In some cases, this led to patients questioning their clinical diagnosis (45). A similar finding was reported by Burns et al. in 2017 who identified patients with hypertrophic cardiomyopathy (HCM) who received a VUS often questioned the validity of their diagnosis and struggled to effectively communicate the familial implications of uncertain genetic test results (46). Further, genetic variants may be re-classified over time, which can also add to the burden of uncertainty for patients (47, 48). Our study adds to this evidence by demonstrating that patients with an uncertain genetic test result have lower perceived self-efficacy compared to those with either positive or negative results. Genetic counsellors are well-suited to support patients’ ability to cope with the complexity and uncertainty of genetic testing results, but are often limited to only one post-test session with patients. The opportunity to meet with a genetic counsellor during follow-up appointments to review their genetic testing results over time may also serve as an intervention to improve self-efficacy. Further research on additional interventions to mitigate the impact of receiving uncertain results on perceived self-efficacy is warranted.

The psychological impact of exercise restrictions has previously been well-described for patients living with inherited cardiac conditions and was further supported by this study (49, 50). ICC clinics should be proactive in identifying those self-limiting exercise due to fear of cardiac symptoms and facilitate access to additional support when needed. Further, care teams are encouraged to use a share-decision making model when discussing exercise restrictions, incorporating the patient's perceived value of exercise to their physical, emotional and social well-being when developing a safe-exercise plan (10, 51).

### Self-reported health-status

In this study, self-reported, mental health scores were associated with perceived self-efficacy and empowerment. Given general self-efficacy and empowerment have previously been correlated with constructs such as anxiety, depression, optimism and shyness, these findings are not particularly surprising, but support the importance of identifying patients with low health status score(s) in order to assess whether any interventions to improve perceived self-efficacy and empowerment are available (17, 25). Systematic psychosocial screening *via* pre-appointment questionnaires have previously been implemented in other out-patient cardiology settings, and may be a useful tool to identify ICC patients and family members with low mental health scores (52). This provides an opportunity to address low score(s) with the families during their appointment, and identify willingness and appropriateness of available interventions, such as referral to psychology services. In this study, only 11% of affected participants and 8% of unaffected relatives reported involvement of a psychologist as part of their care. Improving pathways for patients and their relatives to access these services is recommended for ICC clinics, either by embedding psychology services within a multidisciplinary model or establishing referral process to a psychologist familiar with issues faced by ICC families (53). In Canada, additional barriers to accessing counselling services include financial burden and excessive wait-times, and avenues to reduce these barriers should be considered for ICC patients (52). In addition to psychology services, past research has demonstrated informal peer support opportunities to be desired by cardiology patients, with some evidence supporting this as an effective intervention to improve both self-efficacy and empowerment (54, 55).

### Unaffected relatives and partners

This study found no significant differences in self-efficacy and empowerment scores between affected patients and their unaffected relatives or partners, suggesting family members of those with an ICC may also experience negative psychosocial impacts. Interestingly, access to a heart rhythm specialist was associated with significantly lower self-efficacy scores in unaffected relatives. This finding suggests the act of undergoing cardiac screening and/or being evaluated in an ICC may in of itself reduce general self-efficacy in relatives, even when the results are reassuring. Interestingly, a recent study by Fusco et al. found more than half of ICC relatives (54%) of who tested negative for a familial variant continued to undergo longitudinal cardiac surveillance, which may extend the negative psychological impact of cardiac evaluation (56). Future research evaluating longitudinal psychosocial outcomes in unaffected relatives and interventions to mitigate undue distress is warranted.

## Study limitations

The conduct of community-based research presents significant challenges. Despite best efforts to engage patients and families across Canada, participants from select provinces were over-represented. Therefore, these exploratory results may not be generalizable across all Canadian ICC patients. Participants’ clinical and risk profiles, including genetic test results, were self-reported and not confirmed with clinical records. Survey participants were primarily female (68%), and well-educated, with over 70% reporting post-secondary education, suggesting a potential response bias and a failure to capture important social determinants. Additionally, it's possible participants with greater self-efficacy and empowerment were more likely to respond to the survey, which may have resulted in biased estimates of self-efficacy and empowerment. Importantly, given the cross-sectional design of this project, we’re unable to determine directionality of the relationship between candidate variables and outcome measures. Lastly, associations between time since diagnosis or last follow-up visit and perceived self-efficacy and empowerment are unknown and were not evaluated as part of this study.

## Conclusion

This study identified differences in resource availability, clinical history and self-reported health status impact the perceived self-efficacy and empowerment of patients with ICC and their unaffected relatives. Based on a developed conceptual framework, this HiRO Patient-Oriented Research project was strengthened by utilizing a community-based approach with support from patient partners and advocacy groups. Further efforts to increase access to genetic testing *via* multidisciplinary clinics should be made, given the association with patient empowerment. Development of interventions to mitigate the negative impact of uncertain genetic test results on perceived self-efficacy is warranted. Finally, we recommend ICC clinics develop processes to identify patients and their family members at risk of low self-efficacy and empowerment in order to offer interventions, including establishing pathways to access psychology services.

## Data Availability

The datasets presented in this article are not readily available because sharing of participant data is restricted to those with research ethics approvals and data transfer agreements. Requests to access the datasets should be directed to akrahn@mail.ubc.ca.
