# Supplementary material for: Perceived self-efficacy and empowerment in patients at increased risk of sudden cardiac arrest
Source: Front Cardiovasc Med. 2023 May 15;10:955060. doi: 10.3389/fcvm.2023.955060 (PMC10225561; doi:10.3389/fcvm.2023.955060)
Supplement: Supplementary file 1 [file Table1.pdf]

Supplementary Table 1. Self-Efficacy (GSE) and Empowerment (GCOS) scores by Survey Respondent Type

Patient Type	n*	Mean GSE Score (SD)	Mean GCOS Score (SD)	p-value (GSE)	p-value (GCOS)
I am a parent, sibling and/or child of someone who has been told that they have an inherited heart rhythm condition or experienced an unexplained cardiac arrest or sudden unexpected death	44	28.51 (3.58)	115.53 (20.98)	0.8	0.81
I have been told I have an inherited heart rhythm condition or have experienced an unexplained cardiac arrest	103	28.52 (2.62)	117.91 (21.09)		
I am a spouse/partner or close friend of someone with an inherited heart rhythm condition or who experienced an unexplained cardiac arrest or sudden death	7	29.17 (1.17)	121.60 (25.32)		
I have been told that I have an inherited heart rhythm condition or unexplained cardiac arrest AND I am a parent, sibling and/or child of someone who has been told that they have an inherited heart rhythm condition or experienced cardiac arrest or sudden unexpected death	63	28.93 (2.46)	118.71 (21.71)		

**18 of the 235 survey participants declined to provide an answer to this question and were excluded from this analysis*

Supplementary Table 2. Self-Efficacy (GSE) and Empowerment (GCOS) Scores by Diagnosis

	Affected Patients					Unaffected Relative: Family Member's Diagnosis				
Diagnosis:	**n**	**Mean GSE Score (SD)**	***p***	**Mean GCOS Score (SD)**	***p***	**n**	**Mean GSE Score (SD)**	***p***	**Mean GCOS Score (SD)**	***p***
Long QT Syndrome	65	32.86 (5.00)	0.29	122.09 (19.19)	0.34	12	33.00 (4.84)	0.68	128.70 (20.72)	0.1
Unexplained Cardiac Arrest	17	33.47 (6.28)		116.33 (17.56)		6	34.80 (4.44)		113.20 (19.73)	
ARVC	28	31.79 (5.61)		118.08 (25.62)		10	28.88 (8.43)		110.25 (17.95)	
Brugada Syndrome	16	33.00 (6.04)		118.69 (27.10)		2	32.00 (5.66)		146.00 (NA)	
CPVT	6			113.80 (10.89)		2	32.50 (3.54)		106.50 (2.12)	
Other	11	32.00 (6.32)		104.60 (18.54)		0	NA		NA	
I don't know	4	38.25 (1.71)		110.50 (14.53)		4	31.00 (7.39)		94.33 (18.15)	
Sudden Cardiac Death	NA					14	32.75 (3.77)		112.73 (20.27)	

Table 3. Association between Cardiac History and Self-Efficacy (GSE) and Empowerment (GCOS-24) in Affected Patients:

Clinical History	n	Mean GSE Score (SD)			Mean GCOS-24 Score (SD)		
		**Yes**	**No**	**p**	**Yes**	**No**	**p**
Genetic Testing Performed	124	32.68 (5.57)	32.69 (4.84)	0.98	121.14 (20.53)	105.68 (21.69)	0.004*
Exercise Restrictions	70	31.75 (5.91)	33.43 (4.89)	0.07	112.03 (21.22)	123.76 (19.90)	0.001*
Sudden Cardiac Arrest	46	32.86 (5.68)	32.80 (5.17)	0.95	113.77. (16.75)	120.36 (22.74)	0.11
ICD Implanted	67	32.18 (5.98)	33.02 (4.99)	0.37	114.94 (19.14)	120.42 (22.60)	0.15
ICD Shocks	23	30.26 (6.78)	32.88 (5.40)	0.15	108.24 (16.30)	118.31 (19.95)	0.08
Prior Dx Anxiety or Depression	48	31.20 (6.06)	33.58 (4.92)	0.01*	114.30 (22.59)	120.54 (20.39)	0.05*

Supplemental Table 4. Genetic Test Results on Self-Efficacy and Empowerment in Affected Patients

Genetic Testing Result	Mean GSE Score (SD)	p-value (GSE)	Mean GCOS Score (SD)	p-value (GCOS)
The results were unclear (VUS)	28.85 (5.32)	0.01*	121.84 (23.97)	0.72
Positive	33.16 (5.31)		120.55 (19.92)	
Negative	34.13 (4.40)		126.10 (18.39)	
I don't know the results	29.33 (6.28)		126.20 (21.16)	

p-values reported for ANOVA. Tukey HSD: GSE: Positive vs. VUS (p=0.04); Negative vs. VUS (p=0.02) * denotes statistical significance ($p < 0.05$).

Table 5. Reason for Personal Exercise Restriction on Self-Efficacy and Empowerment in Affected Patients.

Reason for Exercise Restrictions	Mean GSE Score (SD)	p-value (GSE)	Mean GCOS Score (SD)	p-value (GCOS)
I am worried exercise may increase my risk of cardiac arrest and/or ICD shock	23.43 (6.79)	0.11	95.71 (15.07)	<0.001*
I have physical limitations as a result of my inherited heart rhythm condition that prevent me from exercising	31.14 (6.47)		117.86 (17.87)	
My healthcare provider advised me to limit my exercise	32.57 (4.69)		124.75 (14.11)	
Other	33.52 (4.03)		121.32 (20.95)	

p-values reported for ANOVA. Tukey HSD: GCOS: Worry about Increased Risk vs. Physical Limitations ($p = 0.03$), Worry about Increased Risk vs. Healthcare Provider Advise ($p = <0.001$), Worry about Increased Risk vs. Other ($p <0.001$) * denotes statistical significance ($p < 0.05$).

Supplemental Table 6. Association between Cardiac History and Self-Efficacy (GSE) and Empowerment (GCOS-24) in Unaffected Relatives.

**Clinical History**	n	**Mean GSE Score (SD)**			**Mean GCOS-24 Score (SD)**		
		**Yes**	**No**	***p***	**Yes**	**No**	***p***
**Personal History:**							
Genetic Testing Performed	16	33.00 (3.61)	33.89 (5.30)	0.32	115.43 (15.25)	116.00 (22.23)	0.54
**Affected Relative w/:**							
Genetic Testing Performed	31	30.43 (8.19)	30.86 (4.14)	0.16	118.22 (22.08)	103.71 (18.27)	0.22
Sudden Cardiac Arrest	11	29.27 (12.61)	32.00 (3.56)	0.35	121.60 (22.08)	125.08 (21.09)	0.91
Sudden Cardiac Death	23	31.55 (4.76)	32.05 (6.30)	0.78	109.21 (20.33)	123.65 (21.00)	0.04*
ICD Implanted	16	28.36 (5.51)	29.31 (0.95)	0.53	117.09 (21.08)	129.00 (19.34)	0.17
ICD Shocks	10	31.75 (9.36)	35.75 (4.03)	0.66	115.86 (22.88)	109.33 (6.81)	0.28

* denotes statistical significance ($p < 0.05$)

Supplemental Table 7. Self-Efficacy and Empowerment Scores by Self-Reported Health Status in Unaffected Relatives.

		General Self-Efficacy (GSE)					Patient Empowerment (GCOS-24)			
**Self-Reported Health Status:**	B	95% CI		F-statistic	*p*	B	95% CI		F-statistic	*p*
**Global Health - Physical**	1.30	-1.73	4.32	0.74	0.39	-10.87	-22.31	0.57	3.70	0.06
**Global Health - Mental**	5.32	3.09	7.56	23.00	< 0.001*	6.73	-3.91	17.36	1.64	0.21
**Perceived Social Support**	0.73	-0.18	1.64	2.64	0.11	5.64	1.79	9.48	8.80	0.01*

* denotes statistical significance ($p < 0.05$)

Supplemental Table 8. Multiple linear regression model for General Self-Efficacy (GSE): Unaffected Relatives

**Unaffected Relatives** (n= 41)		**B**	**95% CI**	
Highest Education: *(High School)*	Some Post-Secondary Education	0.83	-5.79	7.45
	Bachelor's Degree or Higher	-3.36	-8.95	2.23
Main Activity *(Employed or Student)*	Not currently employed	-1.52	-5.81	2.77
Relationship *(Not in a relationship)*	In a relationship	-0.80	-6.12	4.52
Income *(Under $69,000 per year)*	More than $70,000 per year	-0.61	-4.92	3.70
Healthcare Providers	Heart Rhythm Specialist	-2.27	-6.55	2.01
	Genetic Counsellor	0.25	-3.78	4.28
	Family Doctor	-0.85	-4.34	2.64
	Social Worker	-6.09	-13.10	0.91
Genetic Testing in Relative *(I don't know)*	Yes	-1.47	-5.87	2.94
	No	-3.78	-9.33	1.77
Global Health - Mental		5.69	2.27	9.11
MSPSS		-0.21	-1.44	1.01

Variables in brackets denotes the variable used as reference. MSPSS = Multidimensional Scale of Perceived Social Support. *10 of 51 unaffected relatives did not provide an answer for at least one of the predictor variables are were excluded from this analysis (n=41).*

Supplemental Table 9. Multiple linear regression model for Patient Empowerment (GCOS-24): Unaffected Relatives

**Unaffected Relatives** (n= 35)		**B**	**95% CI**	
Highest Education: *(High School)*	Some Post-Secondary Education	-19.38	-62.75	23.99
	Bachelor's Degree or Higher	-24.96	-64.44	14.52
Main Activity (Employed or Student)	Not currently employed	5.75	-17.58	29.07
Relationship *(Not in a relationship)*	In a relationship	10.52	-14.83	35.86
Income *(Under $69,000 per year)*	More than $70,000 per year	11.81	-10.51	34.14
Healthcare Providers	Psychologist	-3.95	-39.59	31.70
	Social Worker	-9.74	-49.39	29.91
Relative's Diagnosis (*I don't know*)	ARVC	18.30	-21.58	58.19
	Brugada Syndrome	27.47	-33.60	88.53
	Long QT Syndrome	25.02	-14.33	64.38
	Sudden Unexplained Death	24.99	-23.23	73.21
	Unexplained Cardiac Arrest	13.62	-32.52	59.75
Relative SCD	Yes (No)	-24.00	-55.60	7.60
Relative ICD	Yes (No)	-10.33	-44.98	24.33
Global Health - Physical		-11.50	-25.89	2.88
MSPSS		2.23	-3.74	8.21

Variables in brackets denotes the variable used as reference. SCD = Sudden Cardiac Death; MSPSS = Multidimensional Scale of Perceived Social Support. *16 of 51 unaffected relatives did not provide an answer for at least one of the predictor variables are were excluded from this analysis (*
