# Supplementary material for: Perceived self-efficacy and empowerment in patients at increased risk of sudden cardiac arrest
Source: Front Cardiovasc Med. 2023 May 15;10:955060. doi: 10.3389/fcvm.2023.955060 (PMC10225561; doi:10.3389/fcvm.2023.955060)
Supplement: Supplementary file 2 [file Datasheet1.pdf]

Predictors of Perceived Self-Efficacy and Empowerment: Survey

PART 1: Candidate Predictor Variables: Patient Characteristics, History and Risk Profile, Self-Reported Health Status, and Formal and Informal Resources

CANDIDATE VARIABLE	RESPONSE OPTIONS
**1. Patient Characteristics**	
Age	*Number: Continuous variable*
Sex/gender	☐ Male<br>☐ Female<br>☐ Other<br>☐ No answer
Province	☐ British Columbia<br>☐ Alberta<br>☐ Saskatchewan<br>☐ Manitoba<br>☐ Ontario<br>☐ Quebec<br>☐ New Brunswick<br>☐ Newfoundland<br>☐ Nova Scotia<br>☐ PEI<br>☐ Yukon<br>☐ Northwest Territories<br>☐ Nunavut<br>☐ I do not live in Canada<br>☐ No answer

CANDIDATE VARIABLE	RESPONSE OPTIONS
What is the highest level of education you have obtained?	High school<br>Some trade, technical, vocational or business college<br>Some community college or CEGEP<br>Some university<br>Diploma or certificate from community college<br>Bachelor's or undergraduate degree or teacher's college<br>Graduate degree (Master's or PhD)<br>Other, please specify: ________<br>No answer
What do you consider to be your current main activity?	Student<br>Caring for family<br>Working for pay or profit<br>Caring for family AND working for pay or profit<br>Recovering from illness or disability<br>Looking for work<br>Retired<br>Other, please specify: _________<br>No answer
Do you live alone?	Yes<br>No<br>No answer<br>If no, how many people live in your household?
If no, how many people live in your household?	*Number: Continuous variable*
Are you:	Single<br>Married<br>Common law<br>Divorced<br>Separated<br>Widowed<br>Other; please specify: __________<br>No answer
Please estimate your household's total annual income before taxes. Again, we want you to know that all of your answers are confidential and will not be used to recognize you.	Less than $39,999 per year<br>Between $40,000 and $69,999 per year<br>Between $70,000 and $99,999 per year<br>More than $100,000 per year<br>No answer
What language do you speak the most at home?	English<br>French English and another language<br>French and another language<br>Another language; please specify: ____________<br>No answer

CANDIDATE VARIABLE	RESPONSE OPTIONS
**2. History and Risk Profile**	
Please select the category that best describes you:	☐ I have been told I have an inherited heart rhythm condition or have experienced an unexplained cardiac arrest<br>☐ I am a parent, sibling and/or child of someone who has been told that they have an inherited heart rhythm condition or experienced an unexplained cardiac arrest or sudden unexpected death<br>☐ I have been told that I have an inherited heart rhythm condition or unexplained cardiac arrest AND I am a parent, sibling and/or child of someone who has been told that they have an inherited heart rhythm condition or experienced an unexplained cardiac arrest or sudden unexpected death<br>☐ I am a spouse/partner or close friend of someone with an inherited heart rhythm disorder or who experienced an unexplained cardiac arrest or sudden death
OPTION 1: If the respondent self-identifies as having an inherited heart rhythm condition or unexplained cardiac arrest:	
Which inherited heart rhythm condition have you been told you have?	☐ Long QT Syndrome<br>☐ Arrthymogenic Right Ventricular Cardiomyopathy (ARVC)<br>☐ Brugada Syndrome<br>☐ Catecholaminergic Polymorphic Ventricular Tachycardia (CPVT)<br>☐ Short QT Syndrome<br>☐ Idiopathic Ventricular Fibrillation<br>☐ Early Repolarization Syndrome<br>☐ Unexplained Cardiac Arrest<br>☐ Other; please specify: ___________________<br>☐ I don't know<br>☐ No answer

CANDIDATE VARIABLE	RESPONSE OPTIONS
How you were first identified to have an inherited heart rhythm condition?	I had symptoms (cardiac arrest, fainting, dizziness, etc)<br>I was diagnosed after a family member was identified to have an inherited heart rhythm disorder<br>I was found to have heart rhythm disorder after tests were done for other reasons (incidental finding)<br>No answer
How old were you when you were told that you have an inherited heart rhythm condition?	*Number: Continuous variable*
Which healthcare providers have you seen as part of your inherited heart rhythm care? Select all applicable services:	Heart Rhythm Specialist<br>Genetic Counsellor<br>Psychologist<br>Family Doctor<br>Research Nurse/Coordinator<br>Pharmacist<br>Social worker<br>Trauma Counsellor<br>Physical therapist/safe exercise specialist<br>Paediatrician<br>Other: please specify: ________
For any healthcare providers listed above that you have not seen, what is the main reason for not seeing these providers?	My healthcare needs are currently met and I do not need access to additional healthcare providers<br>I would like access to additional healthcare providers but they are not available where I receive care<br>I have financial limitations that restrict the healthcare providers I have access to<br>I am too busy to see additional healthcare providers even if I want to<br>No answer
Have you had genetic testing?	Yes<br>No<br>I don't know<br>No answer

CANDIDATE VARIABLE	RESPONSE OPTIONS
If yes, did the test identify a genetic change thought to explain your heart rhythm disorder (ie. Was the test positive?)	Yes<br>No<br>The results were unclear<br>I don't remember my results<br>No answer
If no, why not?	I was never asked<br>Genetic testing is not available where I receive care<br>Genetic testing was negative or uninformative in my family member<br>I declined<br>Other; please specify: _______________<br>No answer
Do you do less exercise because of your inherited heart rhythm condition?	Yes<br>No<br>No answer
If yes, why?	My healthcare provider advised me to limit exercise<br>I am worried exercise may increase my risk of cardiac arrest and/or ICD shock<br>I have physical limitations as a result of my inherited heart rhythm disorder that prevent me from exercising<br>Other; please specify:_______________<br>No answer
Have you ever had a sudden cardiac arrest?	Yes<br>No<br>No answer
If yes, how old were you when it happened?	*Number: Continuous variable*
If yes, was someone with you when it happened?	Yes<br>No<br>No answer
Do you have an implantable cardioverter defibrillator (ICD) for an inherited heart rhythm condition?	Yes<br>No<br>No answer

CANDIDATE VARIABLE	RESPONSE OPTIONS
If yes, how old were you when you received your first implantable cardioverter defibrillator (ICD)?	*Number: Continuous variable*
Have you ever received a shock from your implantable cardioverter defibrillator (ICD)?	Yes<br>No<br>I don't know<br>No answer
If no, have you ever been offered an ICD for an inherited heart rhythm condition?	Yes<br>No<br>No answer
Were you ever diagnosed with anxiety or depression prior to being told that you have an inherited heart rhythm condition?	Yes<br>No<br>I don't know<br>No answer
Have you informed your immediate living family members (parents, siblings and children) of your diagnosis of an inherited heart rhythm disorder?	Yes<br>No<br>No answer
If yes, have all your immediate living family members (parents, siblings, and children) had screening to determine whether they are also affected with an inherited heart rhythm disorder?	Yes<br>No<br>Some of my family members have had screening<br>I don't know<br>No answer
OPTION 2: If the respondent self-identifies as being a family member, spouse or close friend of an individual who has been told they have an inherited heart rhythm disorder or unexplained cardiac arrest or sudden unexpected death	

CANDIDATE VARIABLE	RESPONSE OPTIONS
Which heart rhythm condition has your family member been told they have	Long QT Syndrome<br>Arrthymogenic Right Ventricular Cardiomyopathy (ARVC)<br>Brugada Syndrome<br>Catecholaminergic Polymorphic Ventricular Tachycardia (CPVT)<br>Short QT Syndrome<br>Idiopathic Ventricular Fibrillation<br>Early Repolarization Syndrome<br>Unexplained Cardiac Arrest<br>Sudden Unexplained Death<br>Other; please specify:<br>I don't know<br>No answer
How many family members have been told that they have an inherited heart rhythm condition?	*Number: Continuous variable*
*Please answer the following questions based on your experience with the **first** family member (other than yourself) who was told they have an inherited heart rhythm disorder or experienced an unexpected cardiac arrest or sudden unexpected death*	

CANDIDATE VARIABLE	RESPONSE OPTIONS
Which province does your family member reside?	☐ British Columbia<br>☐ Alberta<br>☐ Saskatchewan<br>☐ Manitoba<br>☐ Ontario<br>☐ Quebec<br>☐ New Brunswick<br>☐ Newfoundland<br>☐ Nova Scotia<br>☐ PEI<br>☐ Yukon<br>☐ Northwest Territories<br>☐ Nunavut<br>☐ I do not live in Canada<br>☐ No answer
How was your family member/ spouse/ friend <u>first</u> identified to have an inherited heart rhythm disorder?<br><br>(Option 1/3/4 skip to Q3)	☐ They had symptoms (cardiac arrest, fainting, dizziness, etc)<br>☐ They experienced a sudden unexpected death<br>☐ They were diagnosed after a family member was identified to have an inherited heart rhythm disorder/ unexplained cardiac arrest<br>☐ They were found to have a heart rhythm disorder after tests were done
How old was your family member when they experienced a sudden unexpected death?<br><br>(Skip to Q7)	☐ *Number: Continuous variable*

CANDIDATE VARIABLE	RESPONSE OPTIONS
In what year did your family member experience a sudden unexpected death?	*Number: Continuous variable*
How old was your family member when they were first told that they have an inherited heart rhythm condition?	*Number: Continuous variable*
Has your family member/spouse/ friend ever had a sudden cardiac arrest?	Yes<br>No<br>I don't know<br>No answer
If yes, how old were they when it happened	*Number: Continuous variable*
If yes, in what year did your family member experience a sudden cardiac arrest?	*Number: Continuous variable*
If yes, was someone with them when it happened?	Yes<br>No<br>I don't know<br>No answer
Did your family member die as a result of their sudden cardiac arrest?<br><br>(If no, skip to Q10)	Yes<br>No<br>No answer
Did your family member have genetic testing as part of an autopsy?	Yes<br>No<br>I don't know<br>No answer
If yes, did the test identify a genetic change thought to explain their sudden unexpected death (ie. Was the test positive?)	Yes<br><br>No<br><br>The results were inconclusive<br><br>I don't know the results

CANDIDATE VARIABLE	RESPONSE OPTIONS
If no, why was genetic testing not done in your family member?<br><br>(End of part 2)	Genetic testing was not offered from autopsy<br><br>There was no sample saved from autopsy for genetic testing<br><br>My family declined genetic testing in my relative<br><br>Other; please specify: ___________<br><br>No answer
Has your family member ever been offered genetic testing?	Yes<br>No<br><br>I don't know<br><br>No answer
If yes, did the test identify a genetic change thought to explain the heart rhythm disorder in your family member/spouse/friend (ie. Was the test positive?)	Yes<br><br>No<br><br>The results were inconclusive<br><br>I don't know their results<br>No answer
If no, why not?	They were never offered genetic testing<br><br>Genetic testing was not available where my family member received care<br><br>My family member declined genetic testing<br><br>Other; please specify: ____________<br><br>No answer
Does your family member do less exercise because of their inherited heart rhythm condition?	Yes<br>No<br><br>I don't know<br><br>No answer

CANDIDATE VARIABLE	RESPONSE OPTIONS
If yes, why does your family member/spouse/friend do less exercise?	Their healthcare provider advised them to stop exercising<br>They are worried exercise may increase their risk of cardiac arrest and/or ICD shock<br>They have physical limitations as a result of their inherited heart rhythm disorder that prevent them from exercising<br>Other; please specify:________________<br>No answer
Does your family member have an implantable cardioverter defibrillator (ICD)?	Yes<br>No<br>I don't know<br>No answer
If yes, how old was your family member when they received their first implantable cardioverter defibrillator (ICD)?	*Number: Continuous variable*
If yes, has your family member ever received a shock from their implantable cardioverter defibrillator (ICD)?	Yes<br>No<br>I don't know<br>No answer
If no, have they ever been offered an ICD?	Yes<br>No<br>I don't know<br>No answer
Were you ever diagnosed with anxiety or depression prior to your family member being told they have an inherited heart rhythm condition?	Yes<br>No<br>I don't know<br>No answer

CANDIDATE VARIABLE	RESPONSE OPTIONS
OPTION 3: If the respondent self-identifies as being a parent, child or sibling of an individual with an inherited heart rhythm disorder or unexplained cardiac arrest or sudden unexpected death	
Please answer the following questions based on your personal healthcare experience:	
Have you Have you had screening to determine whether you are affected with an inherited heart rhythm disorder?	Yes<br>No<br>I don't know<br>No answer
If no, why have you not had screening to determine whether you are affected with an inherited heart rhythm disorder?	I do not want to know this information<br>I do not have access to screening<br>Other; please specify:______________<br>No answer
Have you had genetic testing?	Yes<br>No<br>I don't know<br>No answer
If yes, did the genetic test identify the same genetic change seen in your family member (ie. Was the test positive?)	Yes<br>No<br>The results were unclear<br>I don't remember my results<br>No answer
If no, why have you not had genetic testing?	I was not offered genetic testing<br>Genetic testing is not available where I receive healthcare<br>Genetic testing was negative or uninformative in my family member<br>I declined genetic testing<br>Other; please specify: _______________<br>No answer

CANDIDATE VARIABLE	RESPONSE OPTIONS
Which healthcare providers have you seen as part of your inherited heart rhythm screening? Select all applicable services:	☐ Heart Rhythm Specialist<br>☐ Genetic Counsellor<br>☐ Psychologist<br>☐ Family Doctor<br>☐ Research Nurse/Coordinator<br>☐ Pharmacist<br>☐ Social worker<br>☐ Trauma Counsellor<br>☐ Physical therapist/safe exercise specialist<br>☐ Paediatrician<br>☐ Other: please specify: ________
For any healthcare providers listed above that you have <u>not</u> seen, what is the main reason for not seeing these providers?	☐ My healthcare needs are currently met and I do not need access to additional healthcare providers<br>☐ I would like access to additional healthcare providers but they are not available where I receive care<br>☐ I have financial limitations that restrict the healthcare providers I have access to<br>☐ I am too busy to see additional healthcare providers even if I want to<br>☐ No answer

CANDIDATE VARIABLE	RESPONSE OPTIONS
**3. Self-Reported Health Status [Physical, Mental and Social Self-Reported Indicators]** ***PROMIS Scale v1.2 – Global Health (http://www.healthmeasures.net)***	

CANDIDATE VARIABLE	RESPONSE OPTIONS
**Emotional Distress – Anxiety PROMIS 4a (http://www.healthmeasures.net)**	

CANDIDATE VARIABLE	RESPONSE OPTIONS
4. **Formal and Informal Resources**	
Social Support - The Multidimensional Scale of Perceived Social Support (MSPSS)	

PART 2: Candidate Outcome Variables

OUTCOME VARIABLE	RESPONSE OPTIONS
**4. Self-Efficacy**	
General Self-Efficacy Scale ***http://userpage.fu-berlin.de/health/faq_gse.pdf***	

OUTCOME VARIABLE	RESPONSE OPTIONS
**5. Empowerment – Adapted Genetic Counselling Outcome Scale (GCOS-24)** http://www.geneticsforpatients.org.uk/<br><br>From: McAllister, M., Wood, A. M., Dunn, G., Shiloh, S., & Todd, C. (2011). The Genetic Counseling Outcome Scale: a new patient−reported outcome measure for clinical genetics services. *Clinical genetics*, *79*(5), 413-424.	
