# Supplementary material for: Perceived self-efficacy and empowerment in patients at increased risk of sudden cardiac arrest
Source: Front Cardiovasc Med. 2023 May 15;10:955060. doi: 10.3389/fcvm.2023.955060 (PMC10225561; doi:10.3389/fcvm.2023.955060)
Supplement: Supplementary file 3 [file Table2.docx]

|  | **Affected** *(n= 160)* | | | | | | | **Unaffected Relative/Partner** *(n= 51)* | | | | | | |
| --- | --- | --- | --- | --- | --- | --- | --- | --- | --- | --- | --- | --- | --- | --- |
| **HCP Accessed for ICC care:** | **n (%)** | **Mean GSE Score (SD)** | | | **Mean GCOS Score (SD)** | | | **n (%)** | **Mean GSE Score (SD)** | | | **Mean GCOS Score (SD)** | | |
|  |  | **YES** | **NO** | ***p*** | **YES** | **NO** | ***p*** |  | **YES** | **NO** | ***p*** | **YES** | **NO** | ***p*** |
| Heart Rhythm Specialist | 152 (95%) | 32.87 (5.36) | 29.25 (5.70) | 0.12 | 118.71 (20.52) | 111.89  (30.68) | 0.53 | 30 (59%) | 30.89  (6.05) | 34.41 (3.61) | 0.02* | 114.33  (9.81) | 120.00 (24.22) | 0.46 |
| Genetic Counsellor | 100  (63%) | 32.96  (5.17) | 32.18 (5.85) | 0.42 | 120.46 (20.47) | 114.35  (22.29) | 0.12 | 19 (37%) | 30.22  (6.63) | 33.56 (4.21) | 0.07 | 114.76  (19.87) | 117.33  (22.59) | 0.70 |
| Psychologist | 17  (11%) | 30.92  (7.55) | 32.86 (5.15) | 0.37 | 114.92 (18.86) | 118.61  (21.56) | 0.52 | 4  (8%) | 22.67  (13.01) | 32.90 (4.12) | 0.31 | 94.67  (18.61) | 117.97  (20.74) | 0.15 |
| Family Doctor | 116  (73%) | 32.41  (5.71) | 33.39  (4.51) | 0.29 | 117.82 (22.16) | 119.36  (18.96) | 0.69 | 22 (43%) | 30.45 (6.59) | 33.64 (4.03) | 0.07 | 116.25  (18.44) | 116.29  (24.15) | 0.99 |
| Pharmacist | 45  (28%) | 32.52 (5.38) | 32.73 (5.47) | 0.83 | 119.26  (17.87) | 117.82 (22.62) | 0.70 | 3  (6%) | 23.50  (19.09) | 32.63  (4.38) | 0.62 | 123.00  (36.77) | 115.92  (20.95) | 0.83 |
| Social Worker | 11  (7%) | 32.40 (7.21) | 32.69 (5.30) | 0.90 | 111.80  (14.39) | 118.77 (21.70) | 0.18 | 5 (10%) | 26.75 (3.60) | 32.76  (5.38) | 0.03* | 100.25 (17.84) | 118.00 (21.10) | 0.14 |
| Physical Therapist | 10  (6%) | 29.00  (6.24) | 32.94 (5.29) | 0.08 | 110.50 (19.50) | 118.88 (21.36) | 0.22 | 0  (0%) | NA | 32.22  (5.49) | NA | NA | 116.27  (21.29) | NA |
| Pediatrician | 3  (2%) | 29.33  (5.13) | 32.74  (5.43) | 0.37 | 103.67  (12.22) | 118.58  (21.35) | 0.16 | 5 (10%) | 33.00 (4.08) | 32.15 (5.65) | 0.72 | 120.75  (19.47) | 115.78  (21.67) | 0.66 |
| Trauma  Counsellor | 6  (4%) | 35.33  (4.13) | 32.56 (5.46) | 0.17 | 113.17  (9.97) | 118.48  (21.66) | 0.27 | 1  (2%) | 31.00  (NA) | 32.25  (5.55) | NA | 144.00  (NA) | 115.58 (21.08) | NA |
| Research  Coordinator | 41  (26%) | 32.23  (5.50) | 32.84 (5.41) | 0.55 | 120.53  (21.16) | 117.32 (21.36) | 0.43 | 10 (20%) | 31.00 (3.81) | 32.53 (5.84) | 0.35 | 114.22  (11.38) | 116.84(3.45) | 0.64 |

HCP = Healthcare Provider; ICC = Inherited Cardiogenetic Condition; GSE = General Self-Efficacy Score; GCOS = Genetic Counselling Outcome Score. Six affected participants did not answer which HCPs they accessed for ICC care and were excluded from this analysis. ** denotes statistical significance (p < 0.05)*
